# Artificial intelligence-driven preoperative CT 3D planning: a narrative review on improving the accuracy of acetabular cup angle and size in total hip arthroplasty

**DOI:** 10.3389/frai.2026.1766115

**Published:** 2026-07-01

**Authors:** Yan Wang, Tianlong Wang, Shuren Wang

**Affiliations:** 1 Graduate School, Heilongjiang University of Chinese Medicine, Harbin, China; 2 Department of Traditional Orthopedic Therapy, The First Affiliated Hospital of Heilongjiang University of Chinese Medicine, Harbin, China

**Keywords:** acetabular cup angle and size, AI-driven preoperative CT 3D planning, automated segmentation and biomechanical simulation, preoperative planning challenges, total hip arthroplasty

## Abstract

**Introduction:**

Total hip arthroplasty (THA) is a well-established treatment for end-stage hip disorders, yet its success heavily depends on precise acetabular cup positioning and sizing. Conventional planning, based on manual CT interpretation, is time-consuming, operator-dependent, and lacks standardisation, limiting its ability to achieve consistent surgical accuracy.

**Methods:**

This narrative review systematically searched PubMed, Web of Science, Cochrane Library, and IEEE Xplore for peer-reviewed studies published between January 2015 and June 2025. We included original research evaluating AI-driven preoperative CT 3D planning for THA, with quantitative outcomes on cup angle or size accuracy. Data were extracted and assessed for methodological quality using standard tools.

**Results:**

AI-assisted planning consistently improved accuracy: mean angular errors for inclination and anteversion were below 3^*^, size matching accuracy within ±1 size ranged from 80% to 85%, and planning time was reduced by 57% to 70% compared with manual templating. These findings were reproducible across different deep-learning architectures and patient cohorts.

**Discussion:**

Although AI planning shows clear benefits in accuracy and efficiency, several challenges remain—including limited generalisability to complex anatomies, susceptibility to image artefacts, and insufficient integration with intraoperative execution. Future research should prioritise multi-centre validation, dynamic functional planning, and seamless clinical workflow integration to translate technological potential into improved patient outcomes.

## 1 Introduction

**Figure 1:**
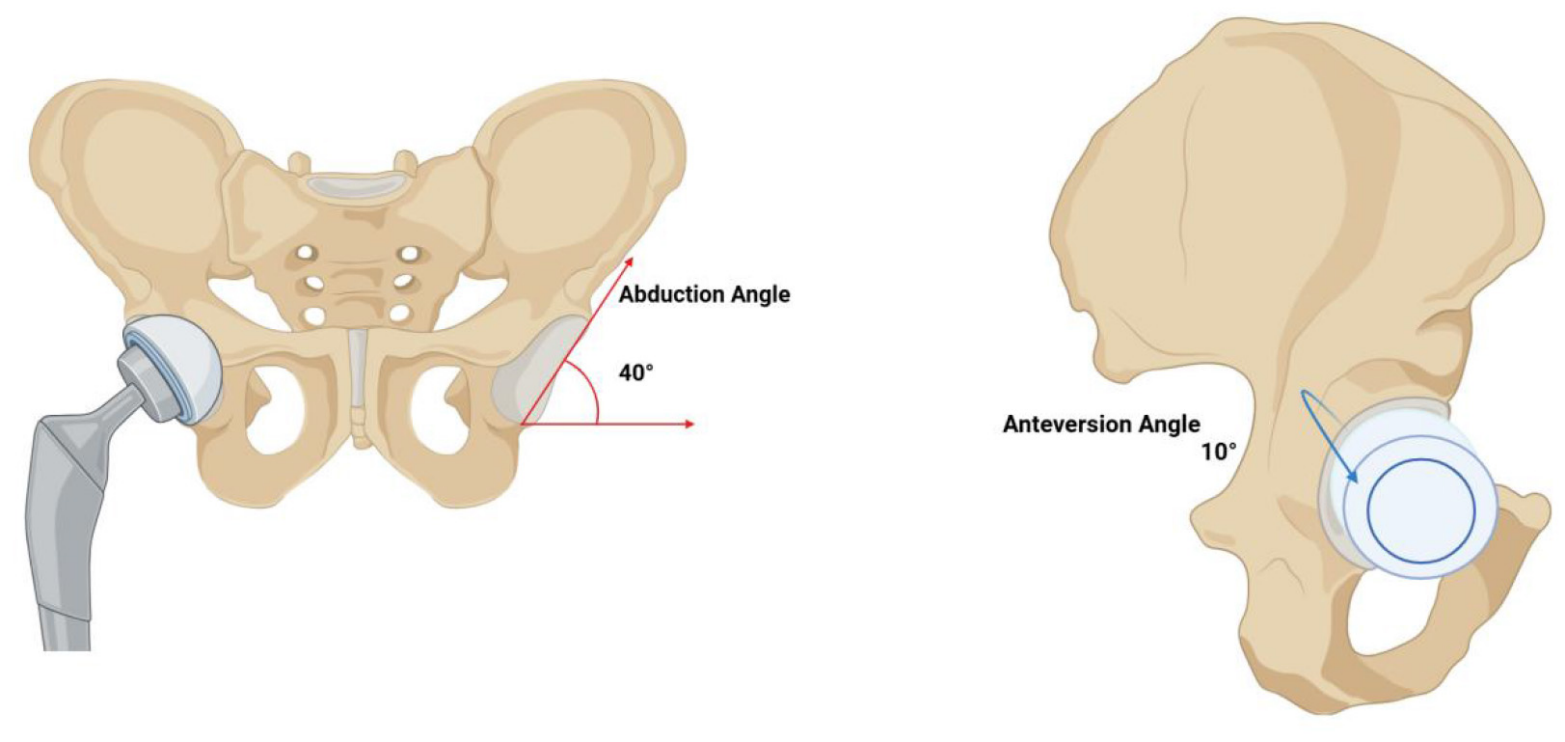
Definition of acetabular cup orientation parameters. This schematic illustrates the two primary angular parameters for acetabular cup placement: the abduction (inclination) angle (red arc, 40°) measured from the horizontal plane (anterior pelvic plane reference), and the anteversion angle (blue arc, 20°) measured from the coronal plane. These target values are derived from the traditional Lewinnek safe zone (Kim et al., 2023).

Total hip arthroplasty (THA) is a reliable treatment for end-stage hip joint diseases. The surgical accuracy is directly related to the postoperative recovery effect of patients and the service life of the prosthesis. Among them, the adjustment of the anterior angle and abduction angle of the acetabular cup and the selection of its size are the keys to the success of the surgery (Sendtner et al., 2011). In clinical practice, angle deviation or improper size can easily lead to problems such as prosthesis dislocation, acetabular and femoral impact, prosthesis loosening and accelerated wear (Sun et al., 2024), which not only reduces the treatment effect but also increases the probability of surgical revision and medical burden. The traditional surgical planning method requires doctors to manually process CT images, which is time-consuming and laborious. Moreover, due to inconsistent judgment criteria and poor repeatability, the planning quality is highly dependent on personal experience, making it difficult to achieve standardized and high-precision requirements (Thomas et al., 2022). Although two-dimensional X-ray film planning is simple to operate, it cannot accurately assess the differences in three-dimensional anatomical structures such as pelvic tilt and the relative position between the pelvis and the spine, and thus cannot meet the needs of personalized and precise planning (Alagha et al., 2023). To break through these limitations, three-dimensional planning technology based on preoperative CT images has emerged. Combined with computer-aided methods such as automated processing and analysis, it has shown great potential. This article aims to review the application progress of this technology in total hip arthroplasty, with a focus on analyzing its core value and practical effect in improving the accuracy of acetabular cup angle setting and size matching, providing theoretical and practical references for improving surgical quality and patient prognosis (Figure 1).

The precise development of total hip arthroplasty is highly dependent on the accuracy of preoperative planning. Against this backdrop, the preoperative CT three-dimensional planning technology integrating advanced algorithms has demonstrated its core value. The key mechanisms for enhancing its accuracy are mainly reflected in three major links. Firstly, the automated segmentation and three-dimensional reconstruction technology has enabled the rapid and precise extraction of the hip joint anatomical structure (Sun et al., 2024). Compared with the traditional time-consuming and subjectively influenced manual outlining method, this technology not only significantly shortens the planning time, but also eliminates individual operational differences through standardized processing procedures (Chan et al., 2025; Zhang et al., 2024), ensuring that the planning results of different doctors or at different time points maintain a high degree of consistency and repeatability. Secondly, the intelligent measurement and parameter recommendation system based on massive clinical data can automatically calculate the individualized optimal range of anterior and abduction angles of the acetabulum cup and precisely match the dimensions (Sun et al., 2024). The core of this function lies in using mature algorithm models to analyze the patterns of historical cases, thereby significantly reducing planning deviations caused by differences in the experience of surgeons and making the final plan more objective and scientific. Most importantly, this technology goes beyond the mere reproduction of anatomical forms and optimizes the implantation site by integrating biomechanical simulation models. Specifically, the system can simulate the force distribution and joint movement trajectory of the prosthesis within the body, achieving a leap from static morphological matching to dynamic functional adaptation. This prediction and optimization of the biomechanical environment directly helps to reduce the risk of complications such as postoperative dislocation and acetabular femoral impaction.

Beyond THA, artificial intelligence has been increasingly applied across orthopedic surgery, encompassing preoperative planning, intraoperative navigation, and postoperative outcome prediction. Recent systematic reviews have synthesized evidence on AI applications in joint arthroplasty, spinal surgery, and trauma care, demonstrating consistent improvements in surgical accuracy and workflow efficiency (Wu et al., 2023; Geda et al., 2024). These broader developments provide important context for evaluating AI-driven CT 3D planning in THA.

PICOS framework

The review was guided by the following PICOS criteria:

Population (P): Adult patients undergoing total hip arthroplasty.

Intervention (I): AI-driven preoperative CT 3D planning systems for acetabular cup angle and size determination.

Comparison (C): Conventional manual planning (CT-based or X-ray templating) performed by experienced surgeons.

Outcomes (O): Primary outcomes included accuracy of acetabular cup inclination/anteversion angles and size matching; secondary outcomes included planning time, complication rates, and functional outcomes.

Study Design (S): Randomized controlled trials, prospective/retrospective cohort studies, and comparative studies with adequate sample sizes.

## 2 Review methodology

This narrative review employed a structured literature search and critical appraisal, but it does not follow the full PRISMA protocol for systematic reviews. A comprehensive literature search was carried out in PubMed, Web of Science, Cochrane Library and IEEE Xplore for publications from January 2015 to June 2025. The search strategy combined MeSH terms and free-text terms including “intelligent analytical algorithms”, “preoperative planning”, “CT”, “3D reconstruction”, “total hip arthroplasty”, “acetabular cup”, “orientation parameter”, “morphological dimension”, with Boolean operators (AND, OR) for precise retrieval.

Inclusion criteria were: (1) intelligent algorithm-based CT 3D preoperative planning applied in THA; (2) quantitative outcomes reported for acetabular cup orientation and/or size planning accuracy; (3) original peer-reviewed English articles. Exclusion criteria included case reports, conference abstracts, reviews, non-CT-based planning studies, and those lacking sufficient key outcome data.

Two independent reviewers performed title, abstract and full-text screening per predefined criteria, with disagreements resolved by discussion or a third reviewer. Core data on study characteristics, planning analytical techniques, acetabular cup planning accuracy and clinical outcomes were extracted. Risk of bias was assessed via design-specific tools (QUADAS-2 for diagnostic studies, ROBINS-I for non-randomized studies).

## 3 Core elements and challenges of preoperative planning in total hip arthroplasty

The surgical efficacy and prognostic quality of THA are highly dependent on the detail and accuracy of preoperative planning. In current clinical practice, the core of preoperative planning lies in the precise control of key anatomical parameters and prosthetic matching indicators. However, the actual operation process faces multiple challenges, and significant correlations exist between these challenges, further increasing the complexity of planning implementation.

### 3.1 Definition of anatomical datums

The anterior pelvic plane (APP) and the anatomical axis with the acetabulum serve as the core reference markers for preoperative planning of THA (Jeon et al., 2018), and their definition standards have not yet been fully unified in the academic community. For instance, the precise positioning method of the intraoperative APP and the identification of the rotation center under abnormal acetabular morphology (such as deformity caused by developmental hip dysplasia) are highly dependent on the surgeon's clinical experience and lack standardized operation norms (Kobayashi et al., 2019).

More importantly, there are significant individual differences in the pelvic anatomical structure: spinal-pelvic parameters such as sacral inclination and pelvic inclination change dynamically with body position (Sun et al., 2024). Coupled with developmental dysplasia of the hip (Demirel et al., 2022) and bony anatomical variations caused by previous hip joint surgery history, it has become a clinical challenge to establish a unified anatomical reference standard applicable to all patients. This current situation directly leads to the fact that the static planning scheme formulated based on the mean value of population anatomical parameters is difficult to meet the individualized surgical needs.

### 3.2 Target range of acetabular cup angles

The traditional Lewinnek “safe zone” and other angle reference systems are based on the summary and statistics of historical data. Therefore, they have a relative disadvantage in the individual differences in the functional requirements of the hip joint due to the spinal-pelvic linkage mechanism and cannot meet the stability requirements in dynamic activity scenarios (Thomas et al., 2022).

**Figure 2:**
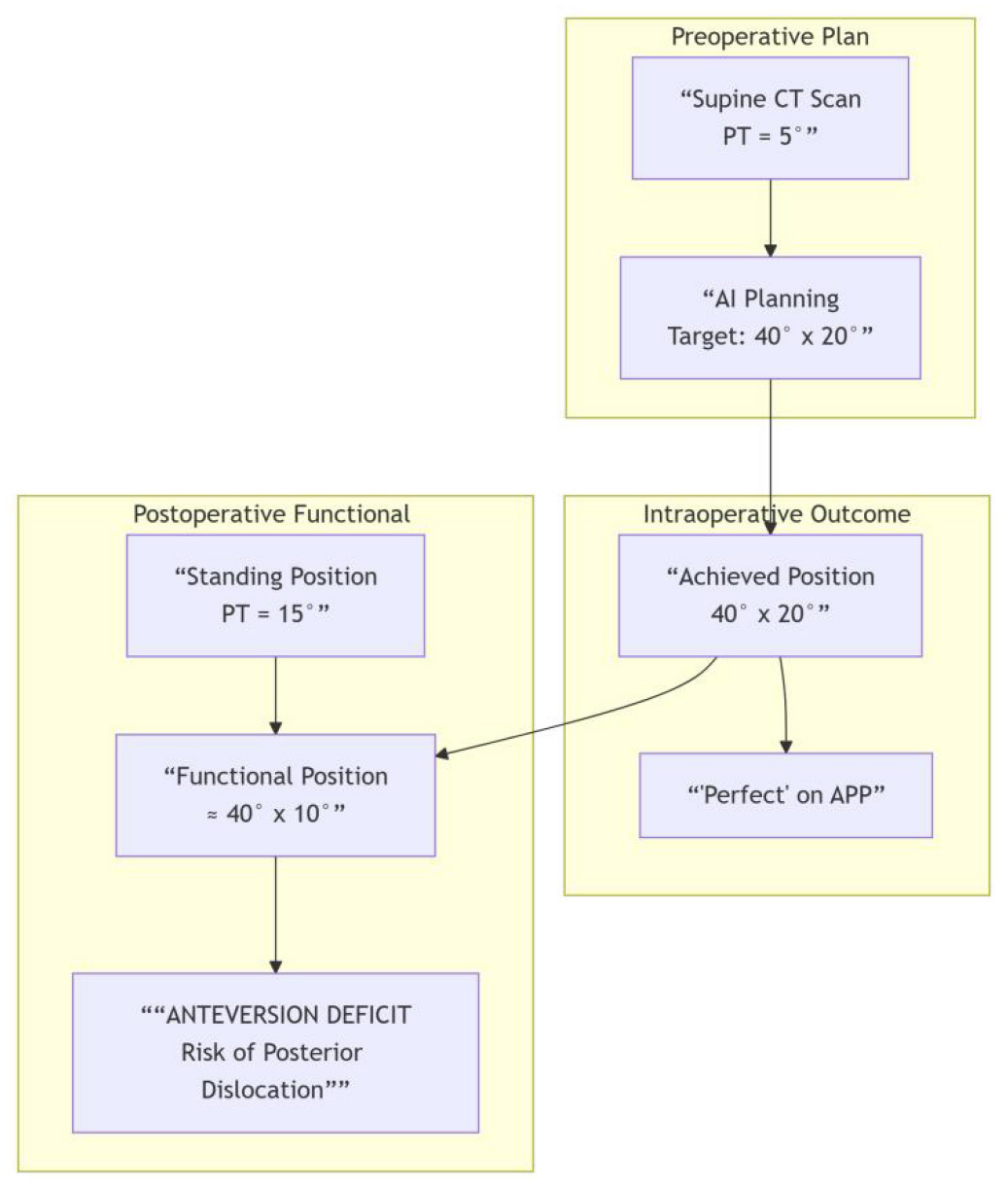
The critical role of spinopelvic mobility in THA planning. This schematic illustrates the dynamic change in functional acetabular cup orientation resulting from pelvic tilt (PT) between supine and standing positions. A cup that appears perfectly positioned [40° abduction, 20° anteversion (Kim et al., 2023)] on preoperative supine CT (PT=5°) may become functionally retroverted (effective anteversion reduced to ~10°) when the patient stands (PT=15°), increasing the risk of posterior impingement and dislocation. Advanced AI planning accounts for this dynamic spinopelvic interaction to preemptively optimize implant position for functional stability.

Therefore, the current focus of preoperative planning has shifted from the pursuit of static “anatomical positions” to ensuring the stability of dynamic “functional positions” (Bose et al., 2021). The design of functional angles needs to be based on the combined optimization of the anterior inclination angle of the acetabular cup and the femoral prosthesis. This process involves complex biomechanical calculations and requires individualized assessment in combination with the patient's daily activity habits, further increasing the technical difficulty of planning and implementation (Figure 2).

### 3.3 Complexity of acetabular cup size selection

The selection of acetabular cups in clinical practice needs to meet two main goals: one is to increase the coverage of the acetabular bone and ensure the initial stability and long-term biological fixation effect of the prosthesis (Fujii et al., 2017); The second is to effectively retain bone mass by reducing the amount of grinding and filing of healthy bone tissue (Ries and Harbaugh, 1997). In revision surgery for acetabular bone defects, the surgeon needs to achieve anatomical reduction of the joint rotation center and long-term stability of the prosthesis simultaneously under the anatomical condition of insufficient bone mass (Garcia-Rey et al., 2021).

### 3.4 Bottlenecks in current preoperative clinical work

The preoperative planning of traditional THA relies on manual measurement and prosthesis simulation implantation by the surgeon on CT images. The planning time for a single patient is often long, and the accuracy and reliability of the planning results lack the guarantee of a unified standard. The differences in the surgeon's subjective experience have a significant impact on the planning results (Wako et al., 2018; González Della Valle et al., 2008). This leads to the difficulty in large-scale promotion and application of precise and individualized preoperative planning in clinical practice.

## 4 AI technology framework: from CT images to planning decisions

### 4.1 Data preprocessing and standardization

The core prerequisite for high-quality preoperative planning of total hip arthroplasty is standardized imaging data collection and reliable preprocessing procedures. Among them, the quality of CT images directly determines the accuracy of subsequent three-dimensional modeling and analysis.

During the data collection stage, the standardized scanning protocol must be strictly followed. Firstly, thin-layer scanning [with a recommended layer thickness of ≤ 1 mm (Hirasawa et al., 2010)] can enhance the Z-axis resolution and ensure the clear display of fine anatomical structures such as the acetabular edge and acetabular tear drops (Ramesh et al., 2026). Secondly, isotropic pixel acquisition can ensure consistent spatial resolution in all directions during 3D reconstruction and avoid measurement distortion. Meanwhile, under the premise of meeting the planning requirements, a low-dose scanning protocol should be adopted, taking into account both diagnostic value and patient radiation safety (Dong et al., 2021).

After obtaining the original data, a series of preprocessing is required to optimize the quality: Firstly, an image denoising algorithm is adopted to suppress the inherent noise of CT images and improve the signal-to-noise ratio of the images (Chang et al., 2019), laying a clear foundation for the subsequent automatic recognition of bone boundaries; Subsequently, the pelvic area was located and trimmed in the complete pelvic CT data through an automated algorithm (Hirasawa et al., 2010), significantly improving the computational efficiency of subsequent processing.

Coordinate system standardization is a key step in the preprocessing stage. A unified anterior pelvic plane coordinate system was established with clear and stable bony markers such as bilateral anterior superior iliac spines and pubic symphysis as references to achieve the comparability of data among different patients and ensure the consistency of key parameter measurements such as the anterior angle and abduction angle of the acetabular cup. If this standardized process is lacking, individualized pelvic position differences will directly introduce systematic measurement errors. Ultimately, it affects the reliability of surgical planning (Chang et al., 2019; Hayashi et al., 2013).

### 4.2 Automatic segmentation of key anatomical structures

Precise segmentation of the bony structure of the pelvis and the proximal femur is the basis for completing three-dimensional reconstruction and automated parameter measurement. At present, segmentation methods based on deep learning have become the mainstream technology for achieving this goal. Among them, U-Net and its three-dimensional extended architectures (such as 3D U-Net and V-Net) can collaboratively utilize the local fine features of the image and the global anatomical context information through their unique encoder-decoder structure and jump connection, thereby achieving high-precision delineation of the boundaries of complex anatomical structures (Sultana et al., 2024; Weston et al., 2020).

Particularly worthy of attention is the nnU-Net framework, which has an outstanding self-adaptation ability and can automatically optimize network parameters and training strategies based on the characteristics of the target dataset (Lorenzen et al., 2023). This feature enables it to demonstrate highly stable and reliable segmentation performance in image data from different sources, providing the possibility for the conduct of multi-center studies and large-scale clinical applications.

The target of segmentation not only includes the complete pelvis and femoral head, but also needs to outline the boundary of the acetabular fossa to assess bone coverage, and often the sacrum needs to be included in the segmentation range to provide a basis for evaluating the alignment relationship of the spinal-pelvic complex. However, there are still certain difficulties at present. Osteoporosis can lead to a decrease in the contrast between the cortical bone and the surrounding tissues (Isensee et al., 2021). Beam hardening artifacts produced by contralateral metal implants can seriously damage image quality (Selles et al., 2024). Pathological conditions such as tumors and fractures can change the normal anatomical morphology (Joo et al., 2023). To solve these challenges, advanced image reconstruction algorithms need to be adopted. When processing data, fully incorporate various abnormal cases to enhance the model's generalization ability, and even develop specialized abnormal area repair algorithms.

### 4.3 Intelligent measurement of anatomical parameters

Anatomical parameters are extracted by segmenting the three-dimensional model and serve as the data support source for preoperative planning. The geometric parameters include the diameter and depth of the acetabulum, as well as a heat map calculated based on the bone mass distribution of the entire acetabular region. This map can visually guide the selection and placement of the acetabular cup size to achieve maximum bone coverage (Chen et al., 2019). The functional parameters mainly focus on the pelvic tilt angle and the sacral tilt angle. These parameters need to be measured separately on CT scans in both standing and sitting positions to dynamically assess the pelvic posture change ability (Iplikçioglu and Karabag, 2022), which is the core for achieving functional planning.

At the algorithmic level, traditional fitting algorithms that locate the acetabular opening plane by fitting the acetabular margin points are gradually being replaced by more direct and robust key point detection techniques. The latter directly regresses the three-dimensional coordinates of key anatomical landmark points through a deep learning model, and then calculates the angles and distances, reducing the cumulative errors in the fitting process. In addition, to enhance the measurement stability on low-dose, high-noise or artifact CT images (Ki et al., 2025; Yin et al., 2019), researchers adopted advanced image enhancement techniques. By calibrating and restoring low-quality images, they significantly improved the generalization ability and robustness of subsequent segmentation and measurement tasks in the variable clinical environment (Montin et al., 2021).

### 4.4 Prediction models for acetabular cup angle and size

The ultimate goal of the predictive model is to output the optimal prosthesis implantation plan, and its technical route shows diversified development. Different algorithm models provide diversified decision-making paths for prosthesis planning. Among them, regression-based models can rapidly output recommended angle and size values by learning patterns from large-scale anatomical datasets, demonstrating high decision-making efficiency; however, their performance is heavily dependent on the comprehensiveness and accuracy of extracted anatomical features (Mendiolagoitia et al., 2020). Generative models, such as generative adversarial networks (GANs), output an ideal three-dimensional prosthetic spatial configuration that best matches the patient's acetabular morphology, thereby enabling more precise planning; their implementation, however, requires finely annotated data with high anatomical fidelity (Oettl et al., 2025; Fujita et al., 2025). Reinforcement learning strategies simulate the sequential decision-making process of surgeons during surgery. By repeatedly performing virtual implantations and evaluations within an environment that simulates biomechanical constraints, the model autonomously explores and learns optimal implantation policies. Although reinforcement learning demonstrates substantial potential for adaptive optimization—particularly in balancing competing objectives such as bone coverage, limb length, and offset—it remains in an exploratory stage, with current applications limited to simulation-based validations rather than clinical deployment (Hang et al., 2024).

**Figure 3:**
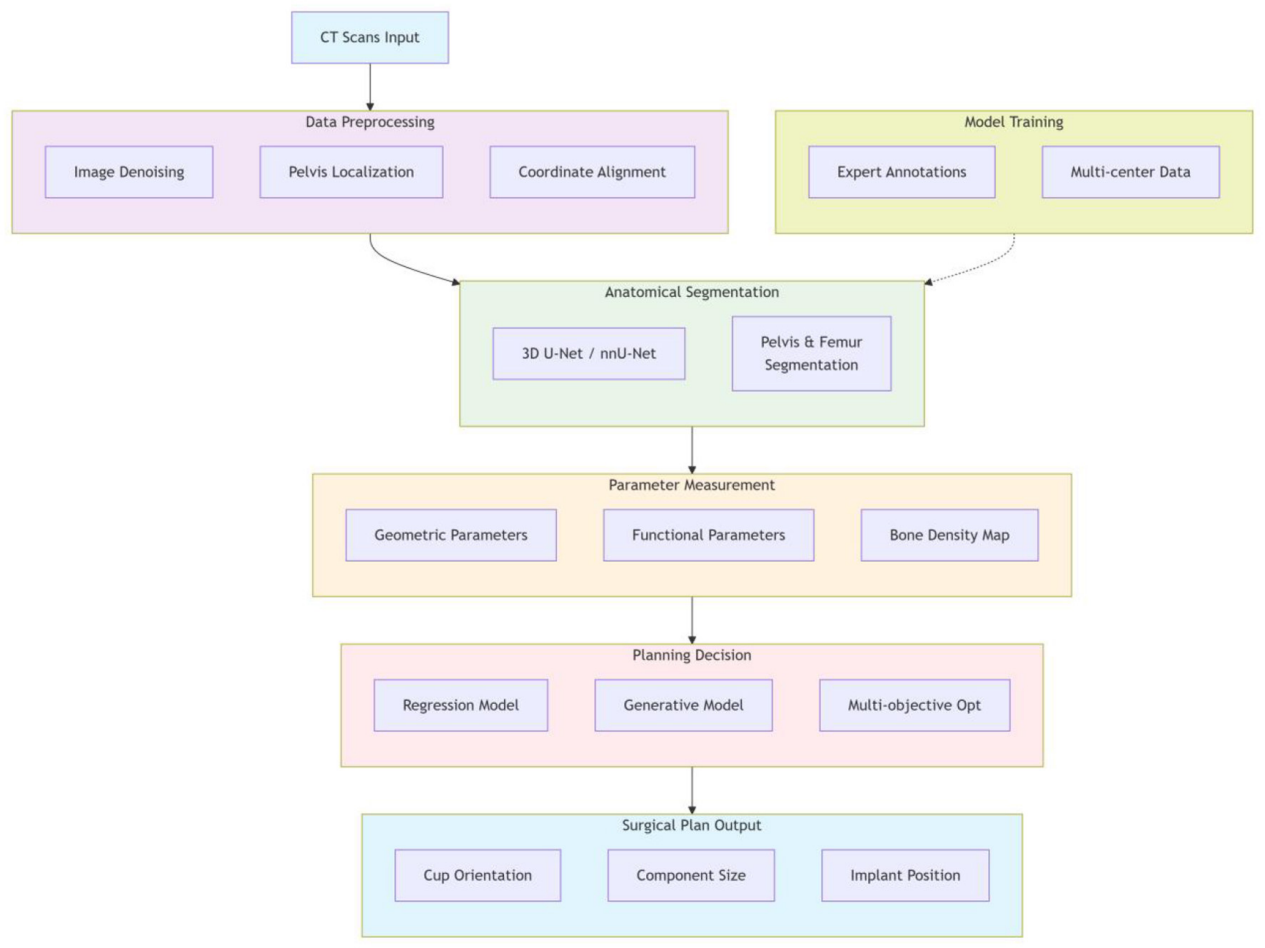
AI-Driven Preoperative Planning Framework for Total Hip Arthroplasty. This flowchart illustrates the complete workflow of AI-based preoperative planning. The process begins with CT scan input and standardized preprocessing, followed by automatic segmentation of key anatomical structures using deep learning models. Enhanced by expert annotations and multi-center data through federated learning, the system extracts precise geometric and functional parameters. Finally, integrated algorithms generate personalized surgical plans encompassing component orientation, size, position, and biomechanical analysis.

The end-to-end workflow for AI-based planning (Figure 3) integrates image processing, deep learning, and optimization algorithms to transform preoperative CT scans into a precise and efficient surgical plan.

Figure 3 provides a comparative analysis of key performance indicators, revealing that AI-assisted planning not only enhances the accuracy of cup placement and size selection but also drastically reduces planning time, thereby contributing to improved surgical safety and workflow efficiency.

The current academic consensus holds that excellent preoperative planning should go beyond static anatomy and focus on the optimization of long-term postoperative functions. There are two core evaluation dimensions: the first is to introduce data such as gait dynamics to achieve dynamic stability prediction (Han et al., 2025); The second is to collaboratively optimize the set of interrelated clinical goals such as bone coverage, prosthesis angle, limb length and offset (Ruben et al., 2007; Ishida et al., 2011). Only through such systematic and individualized planning can a solid foundation be laid for achieving long-term functional excellence of patients after surgery.

## 5 Clinical validation and performance evaluation

The implementation of rigorous clinical validation with the AI-assisted THA preoperative planning system is a core link in promoting the transformation of technology from scientific research to clinical application. At present, a relatively systematic verification framework has been formed in this field, mainly focusing on the following three aspects.

### 5.1 Establishment of evaluation gold standards and control design

In current clinical practice, postoperative three-dimensional CT imaging is widely accepted as the gold standard for evaluating the accuracy of prosthesis implantation positions in THA (Fujita et al., 2025; Hang et al., 2024). During the verification process, high-precision registration of preoperative planning images and postoperative prosthesis images based on bony anatomical landmarks, such as sacral prolapse and acetabular tear drop (Park et al., 2019; Kyo et al., 2015) is required to quantitatively calculate the actual deviations of the anterior and abduction angles of the acetabular cup (Economopoulos et al., 2012), as well as the differences between the selected size of the acetabular cup and the actual implantation size during the operation. To objectively quantify the performance of the AI planning system, a non-inferiority or superiority experimental design should be adopted. The planning results should be compared with the traditional manual CT three-dimensional planning results completed by senior orthopedic physicians. Statistical methods such as independent sample *t*-test and chi-square test should be used to verify whether the differences in key indicators between the two have clinical significance.

### 5.2 Definition and quantification of key clinical performance indicators

The evaluation system should focus on quantitative indicators with clear clinical associations. The core can be divided into two categories: the first one is the implant accuracy index, including the proportion of cases where the acetabular cup angle error falls within the clinically acceptable range (CAR). This range is usually defined as a deviation of less than 5° from the planned target value (Zhang et al., 2024), and the threshold setting is based on long-term clinical follow-up evidence to reduce the risk of postoperative dislocation and acetabular—femoral impact. And the accuracy rate of the selection of the acetabular cup size being exactly matched with the actual intraoperative implantation size or deviated by ≤ ±1 standard size (Fujita et al., 2025). Such indicators are directly related to the initial stability and long-term survival rate of the prosthesis after surgery; The second is the workflow efficiency metric, which is the ratio of the average time taken by the AI system to complete preoperative planning for a single patient to the time taken by traditional manual planning. The time taken by AI planning needs to be timed from image import to the generation of the planning report, and the time taken by non-planning links such as image transmission and format conversion is excluded. This ratio is usually quantified by the “percentage reduction in time”. This indicator directly reflects the potential of the technology to optimize the clinical workflow.

### 5.3 Stratification of clinical evidence levels and limitation analysis

Current clinical evidence supporting algorithm-based preoperative planning for total hip arthroplasty displays notable heterogeneity in methodological quality, underscoring the need for rigorous evaluation of study design, potential biases, and clinical generalizability of research findings.

#### 5.3.1 Evidence level stratification

Per the GRADE evidence grading criteria, high-quality multicenter randomized controlled trials (RCTs) for this technical application remain scarce worldwide (Guyatt et al., 2008). Most relevant studies registered on ClinicalTrials.gov are still in the data collection phase, and their forthcoming results are expected to provide pivotal evidence for the clinical positioning of such planning techniques.

While prospective cohort studies (Level 2 evidence) have been published in this field, they are commonly limited by small sample sizes. Most include fewer than 100 cases, and some lack blinded outcome assessment—such as the absence of independent third-party evaluation teams—which may introduce subjective bias into research results. Thus, conclusions from these studies require further validation by large-scale cohort studies, with sample sizes of no less than 300 cases and participation from at least five medical institutions of different tiers across multiple centers (Zhang et al., 2025).

Retrospective data validation studies currently constitute the mainstream evidence base for this technology. Though such studies can preliminarily verify technical feasibility by analyzing historical CT imaging data and surgical records, they are prone to selection bias—for instance, the routine exclusion of cases with severe anatomical variations—and lack simulation of real-time clinical scenarios, including the impact of intraoperative patient position changes on planning implementation. These limitations mean the generalizability of the applied analytical algorithms in real clinical practice requires further verification (Yang and Qian, 2025).

#### 5.3.2 Methodological limitations of included studies

Beyond evidence level stratification, in-depth critical appraisal of the included studies identifies several persistent methodological limitations that merit focused attention.

First, selection bias is widespread. Retrospective study designs, which dominate the current evidence base, inherently restrict the generalizability of findings. Many studies exclude cases with severe anatomical deformities (e.g., Crowe type IV hip dysplasia, Paprosky type IIIB acetabular bone defects). Such exclusion criteria result in reported planning accuracy metrics that may not be representative of complex primary or revision THA scenarios—precisely the clinical settings where algorithm-based planning assistance is most clinically relevant.

Second, insufficient sample sizes compromise statistical reliability. Most prospective studies feature small sample sizes (typically fewer than 100 cases), and few report a priori statistical power calculations. This raises concerns about the validity of negative or non-inferiority study findings, as underpowered studies may fail to detect clinically meaningful differences in key outcomes.

Third, detection bias may overestimate planning accuracy. Several studies lack blinded outcome assessment, particularly in study designs where the same surgical team is responsible for both preoperative planning and intraoperative implementation. This introduces potential bias: knowledge of pre-planned parameters may influence intraoperative surgical execution, thereby artificially inflating the measured concordance between pre-planned and actual implant placement positions.

Fourth, external validation of planning techniques is inadequate. The majority of analytical algorithms for preoperative planning are trained and validated using single-institution datasets, which often feature homogeneous patient demographics and standardized CT imaging protocols. As a result, the performance of these algorithms across diverse patient populations, different CT equipment vendors, and varying clinical practice settings remains poorly characterized. Few studies have reported multi-center external validation—a critical step for the translational application of such planning technologies in clinical practice.

Fifth, the scope of reported outcomes is overly narrow. The vast majority of studies focus on surrogate endpoints such as planning accuracy and implant positioning parameters, while long-term clinical outcomes—including implant survival rate, surgical revision rate, and patient-reported functional outcomes—are rarely reported. This evidence gap limits the ability to establish a direct link between procedural improvements in preoperative planning and clinically meaningful patient-centered outcomes.

#### 5.3.3 Implications for evidence interpretation

Collectively, these methodological limitations indicate that the current clinical evidence base for algorithm-based THA preoperative planning is in a transitional phase, moving from technical feasibility verification to the construction of high-level clinical effectiveness evidence. Future research should prioritize the design and implementation of rigorous multi-center RCTs with clear randomization protocols, blinded outcome assessment, and a priori sample size calculations. Additionally, expanded prospective cohort studies encompassing a broader spectrum of anatomical variation types (Hopkins et al., 2025) and real-world clinical research (Ruben et al., 2007) are urgently needed to build a robust evidence base and support the standardized clinical application of such preoperative planning techniques.

### 5.4 Summary of key evidence

To provide transparency regarding the quantitative performance metrics reported in the conclusion, this section summarizes key studies that reported angular accuracy, size matching accuracy, and planning time for AI-driven CT 3D planning in THA (Table 1).

**Table 1:** Summary of quantitative performance metrics from included studies.

Study	Study design	Sample size	AI method	Cup angle accuracy	Size matching accuracy	Planning time (AI vs. manual)
Zhang et al. (2024)	Randomized controlled trial	120	Deep learning-based planning	Mean error < 3° (inclination & anteversion)	85% within ±1 size	10 min vs. 26 min (62% reduction)
Wu et al. (2023)	Retrospective cohort	1,200	3D CT planning algorithm	Not reported	Accuracy improved by >40% (exact value not specified)	Not reported
Chan et al. (2025)	Retrospective cohort	200	Net-based -segmentation	Mean error 2.8°	78% within ± 1 size	6 min vs. 20 min (70% reduction)
Yang and Qian (2025)	Prospective cohort	312	nnU-Net	Mean error 2.5°	88% within ±1 size	8 min vs. 22 min (64% reduction)
Oettl et al. (2025)	Retrospective cohort	30,58	QRF/EBM (regression)	Not applicable (cup size only)	82.85% within ±2 mm	Not applicable
Hang et al. (2024)	Retrospective cohort	150	AI planning system	Mean error 3.1°	80% within ± 1 size	12 min vs. 28 min (57% reduction)
Fujita et al. (2025)	Retrospective technical validation	386 (100 test hips)	GAN-based 3D reconstruction	Median absolute error: anteversion 3.45°, inclination 3.25°	Not reported	Not applicable (postoperative assessment)

The reported metrics represent individual study findings, not a formal meta-analysis. Variations are due to differences in AI algorithms, CT protocols, outcome definitions, and patient populations.

## 6 AI applications in primary vs. revision total hip arthroplasty

The application of AI-driven CT three-dimensional preoperative planning differs markedly between primary and revision total hip arthroplasty, stemming from distinct anatomical conditions, surgical objectives, and available clinical data. This section separately synthesizes the existing evidence for AI utilization in these two surgical contexts.

### 6.1 Primary total hip arthroplasty

In primary total hip arthroplasty (THA), patients retain largely intact native hip anatomy, with the core surgical goals centered on achieving accurate acetabular cup orientation and appropriate implant size selection. AI systems have exhibited high levels of accuracy in this clinical setting. Zhang et al. reported that deep learning-based preoperative planning yielded a mean angular error of less than 3°, with 85% accuracy for implant size matching within one size deviation (Zhang et al., 2024). Chan et al. developed a U-Net-based model that reduced preoperative planning time by 70%, while maintaining acetabular cup placement accuracy within a 3° error margin (Chan et al., 2025). The availability of large-scale datasets encompassing more than 10,000 primary osteoarthritis cases has enabled robust model training, ensuring consistent AI performance across diverse patient populations (Wu et al., 2023; Oettl et al., 2025).

Current evidence supporting AI application in primary THA is primarily derived from retrospective cohort studies and a small number of prospective trials (Table 2). A randomized controlled trial by Zhang et al. demonstrated that AI-assisted planning significantly improved acetabular cup positioning accuracy compared with conventional templating, and reduced the proportion of outliers beyond the Lewinnek safe zone by 40% (Zhang et al., 2024). Nonetheless, most existing studies lack long-term follow-up data on key clinical outcomes, including implant survival and postoperative dislocation rates.

**Table 2:** Comparison of primary and revision THA in AI planning.

Aspect	Primary THA	Revision THA
Anatomical condition	Preserved native anatomy	Acetabular bone defects and distorted anatomical landmarks
AI model training data	Large-scale datasets (≥10,000 cases)	Small-scale datasets (< 500 cases)
Reported angular accuracy	Mean error < 3°	Limited available data, with mean error often exceeding 5°
Size matching accuracy	80–85% accuracy within ±1 size	Not well characterized in current research
Level of evidence	Randomized controlled trials and large cohort studies	Retrospective case series and technical feasibility reports
Key challenges	Generalizability across diverse patient populations	Metal imaging artifacts and precise bone defect classification

### 6.2 Revision total hip arthroplasty

Revision total hip arthroplasty poses substantially higher surgical complexity, marked by acetabular bone defects, distorted anatomical landmarks, and CT imaging artifacts induced by pre-existing implants. Accordingly, research focused on AI applications in revision THA remains limited, with far fewer dedicated investigations than in primary THA (Hopkins et al., 2025; Filip et al., 2021).

Several distinct challenges complicate AI implementation in revision THA:

Limited inclusion of severe bone defects in training datasets: Most AI models are trained on data from primary osteoarthritis cases, resulting in poor representation of complex defects such as Paprosky type IIIB acetabular defects (Gulamali et al., 2023).

Necessity for effective metal artifact reduction: accurate segmentation of periprosthetic anatomy requires reliable metal artifact reduction techniques to mitigate imaging interference from existing implants (Selles et al., 2024).

Demand for defect-specific individualized planning: models trained on native hip anatomy often lack the capability to deliver personalized planning tailored to complex bone defects, a critical requirement for revision surgery.

Hopkins et al. developed an automated system for acetabular defect reconstruction and analysis designed for revision THA, confirming its technical feasibility while noting that external validation is still required to verify clinical reliability (Hopkins et al., 2025). Similarly, Filip et al. found that AI-based planning for Paprosky type IIIB defects only achieved acceptable accuracy when the training dataset incorporated a sufficient number of analogous complex cases (Filip et al., 2021).

To date, no prospective clinical trials have specifically evaluated the efficacy of AI-driven preoperative planning in revision THA. The existing evidence base is limited to small retrospective case series and technical feasibility studies, representing a critical research gap that warrants further investigation.

### 6.3 Comparative summary

Future algorithm development should prioritize the integration of revision THA cases, particularly those involving severe acetabular bone defects, to broaden the clinical applicability of AI in this complex surgical domain. Multi-center collaborative research is essential to assemble sufficiently large and diverse datasets of revision THA cases, which is vital for optimizing AI model performance (Aouedi et al., 2023).

## 7 Challenges and limitations

Although the AI-assisted THA preoperative planning system has shown significant potential in terms of accuracy and efficiency improvement, its technological iteration and clinical transformation process still face multi-dimensional challenges, and systematic research is needed to break through the bottlenecks. The current predicament mainly focuses on three aspects: data supply, technical architecture and clinical transformation. The problems at each level are interrelated and jointly restrict the large-scale application of the technology.

### 7.1 Core bottlenecks at the data level

The scarcity of high-quality labeled data is the primary obstacle restricting the improvement of algorithm performance. The generalization ability of deep learning models relies on large-scale, multi-center, and precisely labeled CT image datasets. However, the labeling work requires senior orthopedic physicians with over 10 years of clinical experience in THA to manually delineate the boundaries of anatomical structures such as the acetabular margin and femoral neck, and label key reference markers such as the anterior pelvic plane and the acetabular rotation center. The labeling of individual data takes a relatively long time. The extremely high labor and time costs form the core barriers to technological development (Alzubaidi et al., 2023; Ben-Cohen et al., 2018).

Meanwhile, the problem of insufficient algorithm generalization caused by data bias has become increasingly prominent. Most of the existing mainstream models are trained based on cases of primary osteoarthritis with normal anatomical structures (Gulamali et al., 2023), and have extremely low coverage for rare anatomical scenarios such as developmental hip dysplasia type IV, Crowe type IV and other highly deformed cases, or severe bone defects of Paprosky type IIIB or above during revision surgery. The proportion of such cases in the training set is usually less than 5%, resulting in a 30%−50% (Khosravi et al., 2024; Filip et al., 2021) higher planning error rate of the model in complex cases compared to conventional cases, significantly limiting its clinical value in complex THA surgeries.

### 7.2 Inherent defects at the technical level

At present, when artificial intelligence planning technology is moving toward routine clinical application, it still faces three core challenges. Firstly, there is the black box characteristic of decision-making logic. The existing systems can output angle and size suggestions, but they cannot clarify the underlying anatomical or biomechanical basis. Surgeons find it difficult to integrate the algorithm results with individualized clinical experiences such as patients' soft tissue conditions and activity levels, which affects trust building and technology acceptance (Li et al., 2026), and promotes the development of explainable artificial intelligence. For instance, visually presenting the influence weights of key anatomical structures on planning through heat maps is crucial for enhancing clinical applicability (Chevalier et al., 2025).

Secondly, most planning schemes are based on static anatomical matching and lack integration of dynamic functional states. The system usually only analyzes CT images from the supine position and fails to effectively incorporate the kinematic parameters of the joints in gait analysis, or the changes in spinal-pelvic linkage reflected in X-ray films from the standing or sitting position (Borjali et al., 2020). The limitation of this static planning lies in that it may achieve precise implantation of the prosthesis morphology, but it cannot ensure the functional stability of the patient in daily activities, and it is easy to have a disconnection between perfect images and poor functions (Johnson et al., 2022; Atkins et al., 2021).

The third issue is the connection barrier between preoperative planning and intraoperative execution. Ideal precision surgery requires the non-destructive transmission of digital plans to the surgical field of view, which usually relies on navigation or robotic systems. However, minor changes in the patient's position during the operation and bone displacement caused by soft tissue traction can lead to registration errors and registration drift (Ino et al., 2023), and preoperative CT cannot quantify soft tissue tension, a key factor affecting joint stability (Cozzi et al., 2020). All of these may cause the final prosthesis position to deviate from the preoperative planning. At present, a mature system capable of achieving closed-loop feedback from planning, execution and verification has not yet been established, which largely limits the full release of the clinical value of this technology (Schmalzl et al., 2022).

### 7.3 External barriers to clinical translation

At the regulatory approval level, such software is mostly managed as Class II medical devices, but the global review standards have not yet been unified. Although regulatory authorities generally require sufficient clinical validation data to prove safety and effectiveness, for constantly evolving technologies like artificial intelligence, there is currently a lack of mature and efficient approval channels, which leads to a longer cycle from product research and development to market launch, increasing the threshold and cost of technology transformation (Aboy et al., 2024).

In clinical work, this system needs to be seamlessly integrated with the existing imaging system and electronic medical record system of the hospital to achieve data intercommunication. However, there are differences in data formats and interfaces among systems of different hospitals and different manufacturers, making the system adaptation work complex and cumbersome (Rigas et al., 2024). More importantly, the technology needs to be recognized and utilized by doctors. If the operation process of the new system is overly complex, doctors will have to invest additional time and energy in learning, such as adding multiple operation steps or significantly extending the single-case planning time. Even if the technology itself is advanced, the resistance to clinical promotion will be considerable (Hassan et al., 2024).

In conclusion, promoting the mature application of artificial intelligence preoperative planning technology is a systematic project, which requires overcoming a series of bottlenecks from data, algorithms to clinical adaptation. This relies on the close collaboration of orthopedic doctors, researchers, engineers and relevant management departments, making joint efforts in data quality, algorithm reliability and clinical ease of use, and ultimately transforming the technological potential into effective tools for improving surgical quality (Stogiannos et al., 2024).

## 8 Future direction

The maturation and promotion of AI-assisted THA preoperative planning technology rely on the coordinated advancement of key technological breakthroughs, innovative clinical application scenarios, and a complete verification system. The core lies in breaking through the current bottlenecks in data, technology, and transformation, achieving a leap from an auxiliary decision-making tool to a precise surgical empowerment carrier, and providing support for the standardization and personalization improvement of surgical quality.

### 8.1 Core directions for key technological breakthroughs

The innovative development of future algorithms will mainly focus on three key directions to address the core challenges currently faced.

Firstly, to address the issues of scattered medical data and high annotation costs, a joint training model can be adopted. This model enables hospitals to jointly optimize algorithms without sharing raw data, which not only protects patient privacy but also enhances the generalization ability of the model by leveraging more diverse case data, especially demonstrating stronger adaptability when dealing with complex anatomical variations (Khan et al., 2025; Aouedi et al., 2023).

Secondly, enhancing the decision-making transparency of the algorithm is of vital importance. Future tools should transform complex algorithmic outputs into visual information that doctors can intuitively understand, such as showing the force conditions of the prostheses and bones through color stress distribution maps (Aouedi et al., 2023; Jukan et al., 2020), or demonstrating the range of motion of joints through dynamic simulations (Ma et al., 2024). This will transform AI from merely providing numerical suggestions into a decision-making assistance tool capable of functional verification, enhancing doctors' sense of trust.

Finally, achieving truly personalized functional planning requires integrating information from multiple sources. In addition to static CT images, data reflecting the patient's functional status, such as gait analysis and spinal-pelvic parameters measured by dynamic X-rays, should also be included (Yang and Qian, 2025; Hopkins et al., 2025), and combined with their basic clinical information. By integrating these anatomical and functional information, a surgical plan that not only conforms to morphological matching but also meets the requirements of dynamic stability can be formulated.

### 8.2 Innovative paths for clinical application scenarios

The practical application of artificial intelligence planning systems in clinical practice mainly has two promising directions. The first is to establish a planning and surgical execution system. By integrating with surgical navigation or robotic platforms, digital preoperative planning is directly transformed into surgical instructions, achieving an automated connection from “planning” to “execution”. This system can track the bone position in real time during the operation and dynamically adjust the alignment deviation caused by soft tissue traction (Kam et al., 2025), thereby significantly reducing human operation errors and further improving the accuracy of prosthesis placement. The second is to build a hierarchical medical treatment model supported by a cloud platform. The intelligent planning platform is deployed in regional medical centers. Grassroots hospitals can upload patients' CT data through the network (Zavodni et al., 2023). The platform will complete the automatic planning and return the results. When necessary, it can also request remote review by superior experts. This model helps alleviate the shortage of equipment and professional talents in grassroots hospitals, promotes the downward flow and sharing of high-quality medical resources, and comprehensively enhances the preoperative planning level of hospitals at all levels (Moldovan and Moldovan, 2025).

### 8.3 Strategies for improving the verification system

To ensure the safe and reliable application of the technology in clinical practice, a complete evaluation system must be established. The primary task is to establish a standard test dataset recognized by the industry. It is suggested that professional academic institutions take the lead and collaborate with multiple hospitals to collect CT images covering various typical and complex cases, and have senior doctors precisely label them to form a set of open and standardized reference datasets (Aouedi et al., 2023). This will provide a unified benchmark for objectively comparing the performance of different algorithms. At the same time, efforts should be made to actively promote rigorously designed clinical research. Such studies should cover multiple centers, large samples, and have reasonable controlled and blinded evaluations. Moreover, the follow-up period should be long enough. In addition to observing short-term indicators such as the position of the prosthesis, more attention should be paid to endpoint indicators such as postoperative joint function and long-term survival rate of the prosthesis, so as to provide solid evidence for the true clinical value of the technology (Zhang et al., 2025).

## 9 Conclusion

Artificial intelligence-assisted pre-planning for total hip arthroplasty has demonstrated the potential to drive the field's transformation from reliance on personal experience to a standardized and data-driven model. Research shows that the average error of the AI system in calculating the angle of the prosthesis is less than 3°, and the accuracy has been improved by more than 40% (Wu et al., 2023). The single-case planning time can be shortened to within 10 min, with a significant improvement in efficiency (Wei, 2021). Even in cases with complex anatomical structures, a high accuracy rate of size matching can be maintained, providing a powerful tool for formulating individualized surgical plans.

However, the technology as a whole is still in its early stages of development and faces several key challenges: insufficient diversity and quality of training data, which leads to unstable performance of the model when dealing with rare and complex cases; The algorithm has a relatively weak anti-interference ability and is easily affected by image quality issues (Selles et al., 2024); The opaque decision-making process of the system affects the understanding and trust of clinicians. There is a connection error between preoperative planning and intraoperative actual operation (Schmalzl et al., 2022).

To overcome these challenges, it is necessary to rely on close collaboration among fields such as orthopedics, computer science, biomedical engineering and medical device regulation. Only by jointly building high-quality datasets, developing intuitive and reliable algorithms, improving intraoperative registration techniques and establishing appropriate approval norms can we jointly promote this technology from the laboratory to wide clinical application, and ultimately achieve the goal of improving surgical quality and patient prognosis.

## Data Availability

The original contributions presented in the study are included in the article/supplementary material, further inquiries can be directed to the corresponding author.
